# Blood Pressure Management After Endovascular Thrombectomy

**DOI:** 10.3389/fneur.2021.723461

**Published:** 2021-09-03

**Authors:** Teng J. Peng, Santiago Ortega-Gutiérrez, Adam de Havenon, Nils H. Petersen

**Affiliations:** ^1^Department of Neurology, Yale University School of Medicine, New Haven, CT, United States; ^2^Department of Neurology, University of Iowa Carver College of Medicine, Iowa City, IA, United States; ^3^Department of Neurology, University of Utah School of Medicine, Salt Lake City, UT, United States

**Keywords:** blood pressure, thrombectomy, stroke, cerebral autoregulation, neurocritical care

## Abstract

Endovascular thrombectomy (EVT) has changed the landscape of acute stroke therapy and has become the standard of care for selected patients presenting with anterior circulation large-vessel occlusion (LVO) stroke. Despite successful reperfusion, many patients with LVO stroke do not regain functional independence. Particularly, patients presenting with extremes of blood pressure (BP) or hemodynamic variability are found to have a worse clinical recovery, suggesting blood pressure optimization as a potential neuroprotective strategy. Current guidelines acknowledge the lack of randomized trials to evaluate the optimal hemodynamic management during the immediate post-stroke period. Following reperfusion, lower blood pressure targets may be warranted to prevent reperfusion injury and promote penumbral recovery, but adequate BP targets adjusted to individual patient factors such as degree of reperfusion, infarct size, and overall hemodynamic status remain undefined. This narrative review outlines the physiological mechanisms of BP control after EVT and summarizes key observational studies and clinical trials evaluating post-EVT BP targets. It also discusses novel treatment strategies and areas of future research that could aid in the determination of the optimal post-EVT blood pressure.

## Introduction

Large vessel occlusions (LVO) account for approximately one third of ischemic strokes but contribute to more than half of all stroke-related mortality and severe disability (1–3). Over the last several years, LVO strokes are increasingly recognized as unique entities with distinct cerebrovascular pathophysiology and treatment strategies. Acute stroke interventions have traditionally focused on the rapid recanalization of the occluded vessel to restore blood flow to the ischemic tissue. This may be achieved, in a small percentage, by administering intravenous alteplase or, much more effectively, with the use of endovascular thrombectomy (EVT) (4–6). Despite successful reperfusion, LVO stroke patients may continue to have infarct growth, and half do not regain their functional independence, evidencing a need for adjunctive therapies to further improve outcomes (7, 8). Recent evidence suggests that hemodynamic management may play an important role in post-EVT care. While optimal BP targets remain unknown, clinicians must balance the need for sufficient post-thrombectomy blood flow to prevent further infarct growth with the risk of reperfusion injury and hemorrhagic transformation.

This narrative review aims to describe the physiological mechanisms of how BP influences outcome after EVT, provide an overview of observational studies, and discuss completed and ongoing clinical trials of BP management after EVT. We will also discuss recent advances in personalized blood pressure management approaches and suggest potential areas of future research.

## Cerebral Autoregulation and Physiologic Considerations of BP Management After EVT

To understand the importance and relevance of blood pressure in acute ischemic stroke, it is helpful to review normal brain vascular physiology and how it can become impaired during ischemia. In a healthy brain, cerebral blood flow (CBF) depends on the pressure gradient between the cerebral arteries and veins. The cerebral arterial pressure is equivalent to the systemic arterial blood pressure (ABP), while the cerebral venous pressure is equivalent to the intracranial pressure (ICP) (9, 10). Cerebral autoregulation is the process in which the brain is able to maintain a steady CBF despite fluctuations in ABP and ICP (9, 11). The mechanisms of autoregulation are not entirely understood but have been shown to involve changes in arteriolar diameter, which directly affects cerebral vascular resistance (CVR) (11). CBF is directly related to ABP and inversely related to CVR, such that *CBF* = *ABP/CVR* (12, 13).

Following vessel occlusion, there is a reduction in CBF to the dependent vascular territory. Below a critical threshold of ~10 ml/100 g brain tissue/min, disruption of ion homeostasis and membrane depolarization lead to a loss of neuronal electrical activity (14, 15). Unless blood flow is rapidly restored, the damage becomes irreversible, and cell death occurs. Surrounding the infarct core, there is a zone of hypoperfused, functionally impaired but still viable tissue known as the “ischemic penumbra” (14, 16). The low perfusion pressure in the penumbra creates a pressure gradient that promotes retrograde flow to the ischemic territory (17). The rate of ischemic core growth depends on the duration of ischemia and the degree of collateral blood flow (18).

Ischemia-induced vasoparalysis in the penumbra leads to loss of intrinsic autoregulatory function (19, 20). The decrease in downstream perfusion pressure distal to the occluded vessel, with compensatory dilation of brain arterioles, further impairs autoregulation and causes the CBF to become passively dependent on the ABP (21). The loss of autoregulation after LVO can persist even after revascularization is achieved, rendering the brain vulnerable to fluctuations in perfusion pressure (22, 23). Low ABP can exacerbate cerebral hypoperfusion and cause further ischemic core growth, particularly among patients with incomplete reperfusion. In contrast, elevated ABP may cause excessive flow, cerebral edema, and hemorrhagic conversion (22, 23). Advanced age, greater stroke severity, hyperglycemia, large infarct volume, treatment with alteplase, and poor collateral status have been associated with the development of hemorrhagic transformation and may further increase the risk of reperfusion injury with elevated BP (24, 25). After thrombectomy, the optimal BP requires a careful balance of these opposing concerns to provide the ideal environment for penumbral recovery while avoiding secondary injury from hypo- or hyperperfusion.

Targeted BP therapies aim to maintain BP below or above a fixed threshold. However, BP is one of the most dynamic physiologic variables with significant changes during the early phase after LVO stroke. Sustained increases in BP variability (BPV) may reflect alterations in the mechanisms responsible for cardiovascular homeostasis and represent a potentially modifiable contributor to end-organ damage and poor neurological recovery after LVO stroke (26–30). Although the exact mechanism by which BPV may exert a negative influence remains unknown, direct exposure of the vulnerable oligemic brain tissue to perfusion fluctuations created by elevated systemic BPV may play a critical role. Short-term BP fluctuations can occur due to various internal and external factors, including central sympathetic drive, arterial tone, cardiopulmonary reflexes, humeral mechanisms, blood viscosity, volume status, and medications (31).

## Relevant Observational Studies

Current guidelines by the American Heart Association released in 2019 recommend maintaining a BP goal of ≤ 180/105 for the first 24 h after mechanical thrombectomy (32). These guidelines are based on limited evidence and do not account for individual patient factors such as reperfusion status or infarct volume. Sustained elevations of systolic blood pressure (SBP) have been associated with higher rates of symptomatic intracerebral hemorrhage (sICH) and poor functional outcomes (33–36). The Blood pressure After Endovascular Stroke Therapy (BEST) multicenter prospective cohort study of 485 patients found that a peak post EVT SBP of >158 mmHg best discriminated between favorable (mRS 0–2) from unfavorable (mRS 3–6) (37), confirming the results of a smaller, previously published study (33). To further characterize BP trajectories during the first 72 h after thrombectomy, Kodali et al. used a person-centered modeling approach to identify subgroups of patients with similar BP patterns (38). Patients with high and high-to-moderate SBP trajectories had significantly greater odds of an unfavorable outcome compared to those with persistently low SBP post-EVT.

Several studies have suggested that the optimal BP target may depend on the degree of recanalization (35, 39, 40). Mistry et al. retrospectively evaluated a group of 228 patients with LVO stroke and found that higher peak values of systolic blood pressure in the first 24 h after EVT independently correlated with worse 90-day outcomes (41). Interestingly, hemorrhagic complications, a marker of reperfusion injury, were observed at lower mean peak SBP levels among recanalized patients compared to non-recanalized patients (41). However, others consistently found that the best outcomes in patients with successful recanalization occurred at lower blood pressures (36, 39, 40, 42, 43). Recanalized patients (TICI 2b-3) demonstrate a significant spontaneous decrease in SBP over 24 h after EVT (36). The relationship between post-EVT BP and functional outcomes becomes linear, with the most favorable outcomes at an SBP of 110 mmHg (35, 36). In contrast, non-recanalized patients (TICI 0-2a) show a diminished decline in post-EVT BP, and the relationship with functional outcomes is U or J-shaped. Both high and low average post EVT SBP are associated with worse outcomes.

Whether the association between lower post-EVT BP and better functional outcome in patients with successful recanalization is causal or just a reflection of other stroke-related factors, such as better collaterals, remains unclear. However, physiological consideration and the results mentioned above have led many stroke centers to adopt a tiered approach to BP management after thrombectomy stratified by reperfusion status. An online survey regarding BP management post EVT performed across StrokeNet sites in the United States found that most institutions (39%) used a target SBP of 120–139 mmHg on recanalized patients and permissive hypertension on non-recanalized patients (44). However, the complexity of location of vessel occlusion, degree of recanalization, and final infarct volume suggest that a more nuanced approach may be necessary.

Several observational studies have investigated the effect of differing BP treatment protocols after thrombectomy on radiographic and clinical outcomes. Goyal et al. separated patients into three groups based on post-EVT achieved SBPs of <140, <160, and <180 mmHg. Actual BP targets varied and depended on the treating physician's preference and the patient's clinical status. The investigators found that achieving an SBP goal of <160 mmHg during the first 24 h post-EVT was associated with lower odds of mortality than permissive hypertension (34). This study did not find any significant functional outcome differences at 3 months, but results may have been underpowered due to the small sample size. A more recent large multicenter study by Anadani et al. showed that SBP targets of <140 mmHg after successful reperfusion were associated with lower odds of unfavorable outcome and need for hemicraniectomy than SBP targets of <180 mmHg (36, 42).

Besides BP targets, BPV has emerged as an important aspect of post-EVT hemodynamic management. Studies found that BPV is elevated in patients with larger strokes and associated with higher rates of death and disability in stroke patients that did not undergo EVT (30, 45). Furthermore, antihypertensive drug classes have differing effects on interindividual variation in blood pressure. Calcium-channel blockers such as amlodipine (oral) and nicardipine (intravenous) have been shown to reduce BPV, while b-blocker may increase it (46, 47). Several recent studies have focused on the relationship between BPV and outcome in patients receiving EVT. While there was significant heterogeneity in the frequency and duration of measurements as well as BPV parameters, high BPV was consistently associated with a reduced likelihood of neurological recovery (29, 30, 48).

The effect of reperfusion status on the relationship between BPV and outcome is more controversial. After incomplete reperfusion, higher infarct volumes with surrounding ischemic tissue may make patients more vulnerable to BP fluctuations. Conversely, successful recanalization exposes friable brain tissue to changes in perfusion pressure, potentially increasing the risk of reperfusion injury. While one of the early studies showed the association between BPV and unfavorable outcome appeared strongest among patients with incomplete recanalization (29), others found the opposite effect (49–51). In a *post-hoc* analysis of the BEST study, patients were stratified by recanalization status (TICI 0-2a, 2b, and 3), and high BPV was associated with poor outcomes for patients with TICI 3 exclusively (37). Similarly, another study showed that the effects of higher BPV on outcomes were more pronounced among patients with better reperfusion status (52). A link between higher BP variability and an increased risk of reperfusion injury is supported by Kim et al. They found that high BP variability in the first 24 h following successful EVT was associated with an increased risk of symptomatic intracerebral hemorrhage (53). However, other investigators could not confirm this association (51).

Whether the relationship between high BPV and outcome is causative or an epiphenomenon of stroke-related factors and associated physiologic stressors remains unknown. Chang et al. demonstrated an inverse relationship between BPV parameters and the degree of recanalization (28). They hypothesized that lower recanalization success might increase the chances of insular and adjacent tissue destruction resulting in sympathetic overactivity. However, despite solid conceptual reasons to support a link between BPV and infarct location, other investigators found no association between BPV and injury to the structures of the central autonomic network, arguing against a central origin (51).

While evidence suggests that maintaining a stable BP with low variability after successful thrombectomy may be as important as controlling mean peak BP values, there has not been a clinical trial dedicated to reducing BPV in stroke patients. A summary of observational trials can be found in Table 1.

**Table 1:** Summary of observational studies evaluating post-EVT static BP and dynamic BP (BPV).

**References**	**Sample size and reperfusion status**	**Monitoring duration and frequency of BP recording**	**Blood pressure measurements and comparisons**	**Main results and conclusions**
**Fixed blood pressures thresholds**				
Goyal et al. (34)	217 patients, 67% achieved TICI 2b-3	BP was recorded hourly for 24 h after EVT.	SBP groups of <180/110, <160/90, or <140/90	A 10 mmHg increase in maximum SBP was associated with a lower likelihood of 3-month functional independence (OR 0.70; 95% CI 0.56–0.87, *p* < 0.001) and a higher odds of 3-month mortality (OR 1.49; 95% CI 1.18–1.88; *p* < 0.001). A BP target of <160/90 mmHg was associated with lower mortality than permissive hypertension (OR 0.08; 95% CI 0.01–0.54; *p* = 0.01).
Mistry et al. (41)	228 patients, 76% achieved TICI of 2b-3	BP was recorded hourly for 24 h after EVT.	Mean, maximum, and minimum, SBP	The maximum SBP independently correlated with worse 90-day mRS (OR 1.02; 95% CI 1.01–1.03; *p* = 0.004) and hemorrhagic complications within 48 h (OR 1.02; 95% CI 1.01–1.04; *p* = 0.002).
Goyal et al. (54)	88 patients with TICI 0-2a	BP was recorded hourly for 24 h after EVT.	Mean, maximum, and minimum SBP, and DBP	Maximum SBP was lower in patients with good outcome (mRS 0-2) at 3 months (160 vs. 179 mmHg, *p* = 0.001). Higher maximum SBP was independently associated with lower odds of functional independence (OR per 10 mmHg 0.55; 95% CI 0.39–0.79; *p* = 0.001),
Anadani et al. (55)	298 patients, 92.6% achieved TICI of 2b-3	BP was recorded hourly for 24 h after EVT.	Mean and maximum SBP within 24 h post EVT	Patients with average SBP of <120 mmHg had better 90-day outcomes (median mRS 2 vs. 3, *p* < 0.001) and lower mortality (12 vs. 26%, *p* < 0.01) compared to patients > 120 mmHg. Higher mean SBP was associated with a lower chance of good outcome (OR 0.97; 95% CI 0.94–0.99, *p* = 0.026) No association was found between BP and hemorrhagic transformation.
Anadani et al. (42)	1,245 patients from 10 stroke centers with mTICI score of 2b-3	BP was recorded hourly for 24 h after EVT.	Mean, maximum, and minimum SBP, and DBP	Elevated admission SBP, mean SBP, maximum SBP, SBP range, and SBP SD were associated with increased risk of sICH and the need for hemicraniectomy. Elevated mean SBP, maximum SBP, and SBP range were associated with worse outcomes. Patients with SBP 101–120 mmHg had the highest rate of good outcome (mRS 0-2) and lowest rates of mortality, ICH, and hemicraniectomy.
McCarthy et al. (39)	212 patients, 85.4% achieved TICI of 2b-3	BP was recorded hourly while patients were in the ICU. BP parameters were retrospectively abstracted from the data available in the medical record.	Admission SBP/DBP, peak intraoperative SBP/DBP, daily peak SBP/DBP measured for first 3 days post-EVT	Incremental 10 mmHg increases in peak 24-h SBP were independently associated with increased likelihood of sICH (OR 1.2; 95% CI 1.01–1.49, *p* = 0.048) and a lower probability of functional independence (OR 0.85, 95% CI 0.73–0.98; *p* = 0.031).
Chang et al. (40)	102 patients, 88.2% achieved TICI of 2b-3	After EVT, BP was measured every 15 min for 2 h, every 30 mins for 6 h, then every hour for 16 h.	24-h mean SBP >130 mmHg vs. <130 mmHg	A mean SBP >130 mmHg during the 24 h after EVT was associated with a shift toward a worse outcome on the mRS at 3 months (OR 2.66; 95% CI 1.11–6.41; *p* = 0.03)
Mistry et al. (56)	485 patients from 12 centers, 76% achieved TICI 2b or 3	BP was recorded for 24 h after EVT, frequency of recordings was institution dependent	Maximum SBP	Higher peak SBP associated with poor outcome in unadjusted (OR 1.02; 95% CI 1.01–1.03; *p* < 0.001) but not in adjusted analysis (OR 1.0; 95% CI 0.99–1.01; *p* = 079) A peak SBP of 158 mmHg best discriminated between good (mRS 0-2) and poor (mRS 3-6) outcomes.
Anadani et al. (57)	1,019 patients from 8 stroke centers, with mTICI score of 2b-3	BP was recorded for 24 h after EVT, frequency of recordings was institution dependent	SBP groups of <140, <160, or <180 mmHg	SBP of <140 mmHg was associated with a higher likelihood of good outcome (mRS of 0-2 at 90 days) and a lower likelihood of hemicraniectomy compared to SBP goal of <180 mmHg (OR 1.53; 95% CI 1.07–2.19 and OR 0.18; 95% CI 0.16–0.2, respectively). SBP goal of <160 mmHg was associated with lower odds of 90 day mortality compared to SBP goal of <180 mmHg (OR 0.41; 95% CI 0.18–0.96).
Matusevicius et al. (36)	3,631 patients, 80.4% achieved TICI 2b-3	SBP was recorded before EVT, at the end of EVT, and 2, 4, 12, and 24 h after EVT	Mean SBP and DBP, SBP categorized in 20 mmHg increments	In the TICI 2b-3 group, SBP of >160 mmHg was associated with less functional independence (OR 0.28; 95% CI 0.15–0.53) and increased rates of sICH (OR 6.28; 95% CI 1.53–38.09) compared to the reference group with <120 mmHg. In the TICI 0-2a group, SBP>160 mmHg was associated with an increased likelihood of sICH (OR 6.62; 95% CI 1.07–51.05).
**Blood pressure variability**				
Chang et al. (28)	303 patients, 79.9% achieved TICI of 2b-3	After EVT, BP was recorded every 15 min for 2 h, every 30 mins for 6 h, then every hour for 16 h.	SBP mean, SD, CV, and VIM	BPV parameters (SD, CV, and VIM) over 24 and 48 h decreased with a higher degree of recanalization. Higher BPV was associated with early neurological deterioration and poor functional outcomes at 3 months. (OR range 1.26–1.64; all *p* < 0.05)
Bennett et al. (29)	182 patients, 54.9% achieved TICI of 2b-3	After EVT, BP was recorded every 15 mins for 2 h, every 30 mins for 6 h, then every hour for 16 h.	SBP SD, CV, and SV	Increased BPV parameters (SD, CV, and SV) were associated with a 1-point increase in 90-day mRS (OR range 2.30–4.38; all *p* < 0.002). SV was the strongest and most consistent predictor of worse mRS (OR range 2.63–3.32; all *p* < 0.007)
Kim et al. (53)	211 patients with TICI 2b-3	BP was recorded hourly for 24 h after EVT.	SBP and DBP mean, maximum, minimum, range, SD, CV, SV, and TR	The TR of SBP variation was independently associated with sICH (OR 1.71; 95% CI 1.01–2.89)
Cho et al. (51)	378 patients, 82.8% achieved TICI of 2b-3	BP was recorded hourly for 24 h after EVT.	SBP and DBP mean, SD, CV, and SV	Higher mean SBP and SBP SV during the first 24 h after EVT was associated with a reduced probability of a favorable 3-month outcome (each 10 mmHg increase OR 0.82; 95% CI 0.69–0.97 and each 10% increase OR 0.37; 95% CI 0.18–0.76, respectively). Effects of mean SBP and SBP SV on outcomes were more pronounced on patients with successful reperfusion.
Mistry et al. (37)	443 patients, 88.4% achieved TICI 2b or 3	BP was recorded hourly for 24 h after EVT.	SBP and DBP SD, CV, ARV, SV, and rSD	The highest tertile of systolic BPV (SD, CV, SV, and rSD) was associated with an increased risk of poor outcome (mRS 3-6) and death (adjusted OR range 1.6–2.9, all *p* < 0.05). The association was strongest in patients with complete reperfusion.
Huang et al. (52)	502 patients from 3 stroke centers with mTICI score of 2b-3	BP was recorded hourly for 24 h after EVT.	SBP and DBP SD, maximum, minimum, CV, and SV	Higher CV (OR 1.09; *p* = 0.035), SV (OR 1.08; *p* = 0.004), and SD (OR 1.07; *p* = 0.027) were associated with lower likelihood of a good outcome (mRS 0-2) and increased odds of sICH. The relationship between BPV (SBD SD) and outcome depended on recanalization status.

*OR, odds ratio; CI, confidence interval; mTICI, modified Thrombolysis in Cerebral Ischemia; BP, blood pressure; SBP, systolic blood pressure; SBPV, systolic blood pressure variability; mRS, modified Rankin Scale; sICH, symptomatic intracerebral hemorrhage; DBPV, diastolic blood pressure variability; SITS-TBYR, Safe implementation of treatments in stroke international thrombectomy registry; SD, standard deviation; CV, coefficient of variation; VIM, variation independent of the mean; SV, successive variation; ARV, average real variability; rSD, residual standard deviation; TR, time rate*.

## Clinical Trials Evidence

The recently published Blood Pressure Target in Acute Stroke to Reduce hemorrhaGe After Endovascular Therapy (BP TARGET) was a prospective, randomized, multicenter, controlled, open-label trial aimed to evaluate if BP control with a goal of 100–129 mmHg could reduce the incidence of sICH and improve functional outcomes compared to a goal of 130–185 mmHg in successfully recanalized post-EVT patients (49). A total of 324 patients were enrolled and randomly assigned to either group within 1 h after EVT. Target BP range was maintained for 24 h after EVT. The choice of the antihypertensive agent was at the discretion of the medical provider. To assess the occurrence of sICH, a CT scan was obtained within 24–36 h of EVT. The study did not show any difference in functional outcome or reduction in the rates of sICH with more intensive BP control. Mazighi et al. (50) However, adherence to the assigned BP target was suboptimal, with only 50% of patients in the intense target group achieving the BP goal of 100–129 at 3 h. Moreover, the median percentage of time in range was only 61% for the 130–185 mmHg group and 66% for the 100–129 mmHg group. The study protocol required a time within the assigned BP range of at least 80%. These results indicate that despite setting SBP targets, post-EVT blood pressure may be difficult to control in clinical practice. Furthermore, the frequent administration of antihypertensive medication with more intensive BP control could have induced higher BPV, potentially offsetting the benefits of BP lowering.

Several ongoing prospective randomized control trials are further evaluating the impact of BP after successful EVT. The Blood pressure After Endovascular Stroke Therapy (BEST-II, NCT04116112) is a phase 2 clinical trial that randomly assigns patients with TICI 2b-3 to BP targets of ≤140, ≤160, and ≤180 mmHg. These BPs will be maintained for 24-h post-EVT, and the outcomes measures will be final infarct volume at 36-h and 3-month mRS. The Outcome in patients Treated with Intraarterial Thrombectomy- Optimal Blood Pressure Control (OPTIMAL_BP; NCT04205305) is a clinical trial evaluating the impact of SBP targets <140 vs. <180 mmHg on 36-h sICH, 90-day mRS, and mortality on patients with successful reperfusion after EVT. Similarly, the Second Enhanced Control of Hypertension and Thrombectomy Stroke Study (ENCHANTED 2; NCT04140110) trial will assign patients to an SBP target of <120 vs. 140–180 mmHg during the first 72 h after EVT to evaluate the effect on 90-day mRS. Finally, the Invasive Control of Blood Pressure in Acute Ischemic Stroke After Endovascular Therapy on Clinical Outcomes (CRISIS I; NCT04775147) trial will compare SBP targets of <140 and <120 mmHg on 90-day sICH and mRS. All ongoing clinical trials will evaluate patients with successful EVT with TICI 2b-3, which is achieved in over 80% of patients with modern EVT techniques. The results of these trials will provide much-needed high-quality evidence and inform blood pressure management after thrombectomy. A summary of completed and ongoing prospective studies can be found in Table 2.

**Table 2:** Summary of completed and ongoing prospective studies evaluating post-EVT static BP and dynamic BP (BPV).

**References**	**Year**	**Trial name and type**	**Status**	**Sample size**	**Blood pressure comparisons**	**Outcomes/goals**
Mazighi et al. (49, 50)	2021	BP TARGET; Randomized, controlled, open-label trial. Patients were enrolled at 4 clinical sites.	Completed	324 patients post EVT with TICI 2b-3	Patients were randomized within 1 hour after EVT to BP target of 100–129 mmHg vs. 130–185 mmHg	Primary outcome: Radiographic ICH Secondary outcome: NIHSS at 24 h, and 3-month mRS Results: There was no difference in the rate of radiographic ICH or any of the secondary clinical efficacy outcomes.
PI: Mistry NCT04116112	Estimated completion: 2023	BEST-II; prospective, randomized trial	Ongoing	120 patients post EVT with TICI 2b-3	Assigned to SBP target of <180, <160, or <140 mmHg during first 24 h after EVT	Primary outcomes: Final infarct volume and utility-weighted mRS at 90 days
PI: Nam NCT04205305	Estimated completion: 2024	OPTIMAL BP; prospective, multicenter randomized trial	Ongoing	644 patients post EVT with TICI 2b-3	SBP target of <140 vs. <180 mmHg during the first 24 h after EVT	Primary outcomes: 90-day mRS, symptomatic ICH at 36 h, death at 90 days ASPECTS at 36 h
PI: Song NCT04140110	Estimated completion: 2023	ENCHANTED 2; prospective, randomized trial	Ongoing	2,236 patients post EVT with TICI 2b-3	SBP target of <120 vs. 140– 180 mmHg during first 72 h after EVT	Primary Outcome: 90-day mRS
PI: Zhou NCT04775147	Estimated completion: July 2023	CRISIS I; prospective, randomized trial	Ongoing	500 patients post EVT with TICI 2b-3	SBP target of <120 vs. <140 mmHg during first 72 h after EVT	Primary Outcome: 90-day mRS

*EVT, endovascular thrombectomy; TICI, thrombolysis in cerebral infarction; SBP, systolic blood pressure; BP, blood pressure; SBPV, systolic blood pressure variability; DBPV, diastolic blood pressure variability; BEST, Blood Pressure After Endovascular Stroke Therapy; mRS, modified Rankin Scale; ICH, intracerebral hemorrhage; NIHSS, National Institutes of Health Stroke Scale; DBP, diastolic blood pressure; ASPECTS, Alberta Stroke Program Early CT Score; PI, Primary Investigator*.

## Individualized BP Targets

Despite the consistent findings of better outcomes with lower post-thrombectomy BP, reducing BP to the same fixed target may be an oversimplification of the complex physiology. The neutral results of the BP TARGET trial suggest that stratification by reperfusion status may be inadequate for the heterogenous EVT patient population. A one-size-fits-all approach does not account for individual patient factors such a collateral status, infarct size, or history of hypertension. For example, a patient with chronic hypertension may have a rightward shifted autoregulatory curve (58), and aggressive BP lowering could result in cerebral hypoperfusion and infarct expansion. Similarly, a patient with TICI 2b reperfusion may still have significant residual tissue at risk and thus require different BP targets post-EVT than a patient with TICI 3 reperfusion. In contrast, a patient with a large infarct despite successful EVT who also received intravenous thrombolytics is at increased risk of reperfusion injury related to hyperperfusion of vascular beds with failed autoregulation. This patient may require strict BP control. In clinical practice, not all thrombectomy patients are treated equally, and survey data indicate that physicians often select BP parameters on a per-patient basis (48). While it may be reasonable to aim for a lower BP, particularly after successful reperfusion, the optimal target remains unknown.

Besides maintaining BP below a fixed, predetermined value, there is a strong rationale for a more personalized BP management. We recently compared fixed vs. personalized autoregulation-oriented BP thresholds after EVT (59). Near-infrared spectroscopy-derived tissue oxygenation (NIRS) was used as a surrogate of CBF and compared to changes in mean arterial pressure (MAP) to calculate the autoregulatory index and the BP range at which autoregulation is most preserved. This study showed that exceeding this blood pressure range was associated with an increased risk of hemorrhagic transformation and worse functional outcomes. Using the same dataset, the association was not seen when applying fixed blood pressure thresholds. Currently, there are no clinical trials to support autoregulation-guided BP management as a potential post-EVT neuroprotective strategy. An impediment to testing autoregulation-guided blood pressure management has been the complex task of acquiring, integrating, and real-time processing of high-frequency physiologic data. Commercially available software and hardware solutions are emerging, which may help to implement a personalized approach to BP management in the clinical setting. A phase II clinical trial of autoregulation-oriented BP management after severe TBI has recently been completed, and the results are expected soon. The study will provide further data regarding the feasibility of a personalized BP management approach that would also apply to other forms of acute brain injury such as ischemic stroke.

Another easily implementable and generalizable approach to personalized BP management is currently being tested in the Effect of Individualized vs. Standard Blood Pressure Management during Mechanical Thrombectomy for Anterior Ischemic Stroke Trial (DETERMINE; NCT04352296). The study will compare maintaining the BP within 10% of the first MAP measured in the angiography suite vs. a fixed blood pressure goal of 140–180 mmHg. Selecting BP targets based on an individual patients' baseline BP could represent a valid method to incorporate patient-specific factors without the need for advanced monitoring. A similar approach could potentially also be implemented in the post-EVT setting.

## Future Directions

Management of hemodynamics in the post-EVT patient is complex and requires careful BP control to ensure adequate CBF to supply the ischemic penumbra while avoiding reperfusion injury. Several clinical trials are underway to evaluate different fixed BP thresholds after EVT with successful recanalization. These studies will provide insight and guidance on the optimal post-EVT BP target. Still, future *post-hoc* analyses of these trials will be necessary to assess if stroke or patient characteristics have any effect on outcome.

Patients with high SBP trajectories during the first 24 h after mechanical thrombectomy are at increased risk of an unfavorable outcome. Future research should focus on the early identification of patients with high-risk trajectories that are most likely to benefit from intense post-EVT BP management. Autoregulation-guided blood pressure management may present an elegant alternative over the classical approach of maintaining blood pressure below a fixed, predetermined value. Clinical trials are needed to test autoregulation-based treatment strategies, including tailored pharmacologic blood pressure augmentation and lowering therapies based on patients' real-time autoregulatory status. Parallel lines of inquiry have begun in Europe targeting autoregulation in traumatic brain injury, further suggesting that this approach is timely and feasible in a multicenter clinical trial.

Other approaches to post-EVT BP management include the reduction of potentially harmful BP fluctuations. There is ample evidence showing that higher BPV is associated with worse functional outcomes and increased ICH risk. Although it is unclear if this is causative or an epiphenomenon related to the stroke itself, studies aiming to decrease BPV should be pursued (28, 29, 51, 53). The main challenge of assessing and manipulating BPV is the lack of bedside techniques to measure BPV in real-time and determine ideal candidates for clinical trials. Short-term BP variability has traditionally been measured as minute-to-minute or hour-to-hour oscillations. It requires a minimum 24-h period, which limits its usefulness for bedside clinical decision making (29, 51–53). One promising approach that warrants further investigation is the use of spectral analysis of beat-to-beat blood pressure to detect harmful patterns of BP variability within minutes (60). Ferreira et al. performed 5-min BP recordings recorded using non-invasive finger plethysmography immediately following TICI 2b-3 revascularization and found that a high-frequency BPV was correlated with lower rates of early neurological recovery and worse functional outcomes (60).

Cerebral perfusion optimization in the immediate post-EVT period is a research priority in acute stroke. Current research is focused on elucidating the best hemodynamic biomarker for defining and monitoring the ideal BP target after stroke. Results from ongoing and future clinical trials will be critical to reduce reperfusion injury and maximize neurological recovery for all stroke patients undergoing endovascular therapy.

## Author Contributions

NP conceived the review. TP drafted the manuscript. SO-G, AH, and NP made critical revisions to the manuscript. All authors contributed to the article and approved the submitted version.

## Conflict of Interest

The authors declare that the research was conducted in the absence of any commercial or financial relationships that could be construed as a potential conflict of interest.

## Publisher's Note

All claims expressed in this article are solely those of the authors and do not necessarily represent those of their affiliated organizations, or those of the publisher, the editors and the reviewers. Any product that may be evaluated in this article, or claim that may be made by its manufacturer, is not guaranteed or endorsed by the publisher.
